# Prevalence, consequences, and contributing factors beyond verbal and physical workplace violence against nurses in peripheral hospitals

**DOI:** 10.3389/fpubh.2024.1418813

**Published:** 2025-01-07

**Authors:** Mohammad M. Alnaeem, Khaled Hasan Suleiman, Majdi M. Alzoubi, Yasmeen Abu Sumaqa, Khalid Al-Mugheed, Amany Anwar Saeed Alabdullah, Sally Mohammed Farghaly Abdelaliem

**Affiliations:** ^1^Adult Health Nursing/Palliative Care and Pain Management, School of Nursing, Al-Zaytoonah University of Jordan, Amman, Jordan; ^2^School of Nursing, Al-Zaytoonah University of Jordan, Amman, Jordan; ^3^Faculty of Nursing, Al-Zaytoonah University of Jordan, Amman, Jordan; ^4^Nursing Department, Al-Balqa Applied University, As-Salt, Jordan; ^5^College of Nursing, Riyadh Elm University, Riyadh, Saudi Arabia; ^6^Department of Maternity and Pediatric Nursing, College of Nursing, Princess Nourah bint Abdulrahman University, Riyadh, Saudi Arabia; ^7^Department of Nursing Management and Education, College of Nursing, Princess Nourah bint Abdulrahman University, Riyadh, Saudi Arabia

**Keywords:** workplace, violence, nurses, incident, emergency room

## Abstract

**Background:**

Globally, nearly one-third of workplace violence (WPV) occurs in the health sector. Exposure to WPV among Jordanian nurses has been widely speculated to be underreported. Understanding of the factors contributing to WPV among nurses and their consequences is limited.

**Objectives:**

This study aimed to examine the consequences and contributing factors of WPV and explore suggestions for reducing WPV among nurses working in peripheral hospitals.

**Methods:**

This descriptive, cross-sectional study included 431 Jordanian nurses. Data were collected using a self-report instrument between December 2022 and June 2023. A modified version of the ILO/ICN/WHO/PSI Workplace Violence in the Health Sector Country Case Study Questionnaire developed and validated in 2003 was used.

**Results:**

The ages of the participants ranged from 20 to 49 years. A total of 349 nurses (81%) had experienced verbal violence, while 110 (25.5%) had experienced physical violence. Of the 110 nurses who were physically attacked, 44 (40 %) reported that an investigation was conducted to determine the cause of the incident. Approximately 38.2% of incidents involving physical violence in the last 12 months involved the use of weapons. The current study revealed that 59.6% of the nurses reported that verbal incidents were common in their workplace. The highest level of agreement among all participants was leniency in applying penalties to perpetrators of violence inside hospitals. The majority of participants (95.8%) agreed that improving staff-patient communication skills would effectively reduce violence.

**Conclusion:**

Creating awareness among healthcare professionals, patients, and the general public regarding the impact of WPV and the importance of respect and professionalism is crucial.

## Introduction

Workplace violence (WPV) is defined as “violent acts, including physical assaults and threats of assaults, directed toward persons at work or on duty” (1, 2). Violence can be divided into physical, sexual, psychological, and verbal categories based on the type of activity. It can also be separated based on the sources of violence: internal, which is carried out by the same organization's managers and workers, or external, which is carried out by others, such as clients and criminals (3, 4).

WPV in healthcare settings includes any statement or behavior that gives a worker a reasonable cause to believe they are threatened (3, 5). Nurses are three times more likely to be exposed to violence (6, 7). WPV prevalence among nurses was previously reported to be 43% in the United States (8), 44% in Japan (9), and 67% in Italy (10). The high rate of WPV could make the workplace unsafe and make nurses afraid of experiencing WPV in the future (11, 12). WPV against nurses is a significant global issue that has recently received more attention (13). Over 50% of registered nurses reported experiencing verbal abuse or bullying, while about 25% of them reported that a patient or family member had physically abused them (7, 14–18). In healthcare settings, violence often occurs in complex care environments such as intensive care units and emergency departments. These settings involve urgent and complex care, which can lead to conflicts and misunderstandings between healthcare teams and patients or their families (5, 19, 20). Conflicts may arise from differing views on medical decisions, creating tense emotional states and diverging expectations (14, 19, 21, 22).

According to several studies, violence in Jordanian hospitals negatively impacts healthcare services and personnel stability (23–25). The outcomes demonstrated a high prevalence of physical and verbal aggression toward healthcare providers in Amman's public sector hospitals (16, 26–28). The prevalence of WPV committed against nurses in hospital emergency departments revealed that 76% had experienced some form of violence, with verbal violence being approximately five times more common than physical violence (63.9% vs. 11.9%) (26). Patients committed 7.2% of the violations and visitors committed 3.1% (29, 30). In addition, most studies have discovered that workers who have experienced WPV have significant levels of anxiety, sadness, generalized fear, frustration, insomnia, and emotional issues, which can lead to more serious conditions such as post-traumatic stress disorder or burnout (9, 24, 31). Furthermore, WPV may lead to avoidance behavior, delay in effective communication, impaired peer relations, poor concentration at work, preventing patients from delivering safe and effective nursing care, failure to raise safety concerns, and seeking assistance/delayed care (6, 32–34). Furthermore, job dissatisfaction, increased staff turnover rates, and treatment or medications (35). Many studies have reported that physical WPV can have immediate negative effects including bites, bruises, lacerations, and hair loss (36). The consequences of violence on health organizations are also significant when considering absences due to work injuries or absenteeism, burnout, and decreased job satisfaction, all of which have a significant impact on work quality, budget, and costs (17, 37).

Previous literature has highlighted the worrisome rates of WPV and aggressiveness faced by nurses working in central hospitals compared to peripheral hospitals located outside the capital of Jordan. Peripheral hospitals in rural areas have certain socioeconomic, geographic, and infrastructural characteristics that differ from those in urban areas. They are characterized by lower population densities, agricultural economies, and less developed infrastructure than bustling, urbanized areas (38). While WPV has received significant attention in large central hospitals, the severity and consequences of violence in peripheral (rural) hospitals are frequently neglected or underestimated (39, 40).

Previous research has reported the relationship between WPV and healthcare workers' gender, occupation, practice environments, and work schedules (37, 41, 42). Factors contributing to violence include long wait times for patients, overcrowding in the emergency department, patient and family expectations of medical staff, lack of resources, lack of staff experience, lack of staff attitude, poor management/admission procedures, lack of rules and penalties, public ignorance, and the influence of drugs or alcohol are all possible factors (7, 8, 43–45).

Violence against nurses is still underreported (8, 15, 39, 44). The most common reasons for not reporting WPV were nurses' lack of knowledge about how and what types of violence to report, hospitals' preference for patients over nursing staff, and a lack of supervisory support after reporting (7, 25, 44, 46). A lack of a hospital reporting system could also be a contributing factor (6, 16, 47, 48). This study aimed to examine the consequences and contributing factors of WPV and explore suggestions for reducing WPV among nurses working in peripheral hospitals. Further, this study addresses the following research questions:

1. What are the consequences of WPV against nurses who work in peripheral hospitals?
2. What are the contributing factors beyond WPV among nurses who work in peripheral hospitals?
3. What are the suggestions for reducing WPV among nurses working in peripheral hospitals?

## Methods

### Design

Descriptive cross-sectional design. The study followed the EQUATOR Research Reporting Checklist and the STROBE Checklist for cross-sectional research.

### Settings and sample

This study was conducted at six government hospitals in the peripheral regions of Jordan. A convenience sample of nurses was recruited. The inclusion criteria were nurses working in the emergency department/intensive care units/medical-surgical floors. Nurses in outpatient departments and those with administrative roles were excluded. A total of 700 questionnaires were distributed and 490 were returned (response rate = 70%). Fifty-nine questionnaires were excluded from the analysis because they were incomplete as ≥50% of items were unfinished. The final sample consisted of 431 nurses.

### Data collection procedure

**Figure 1:**
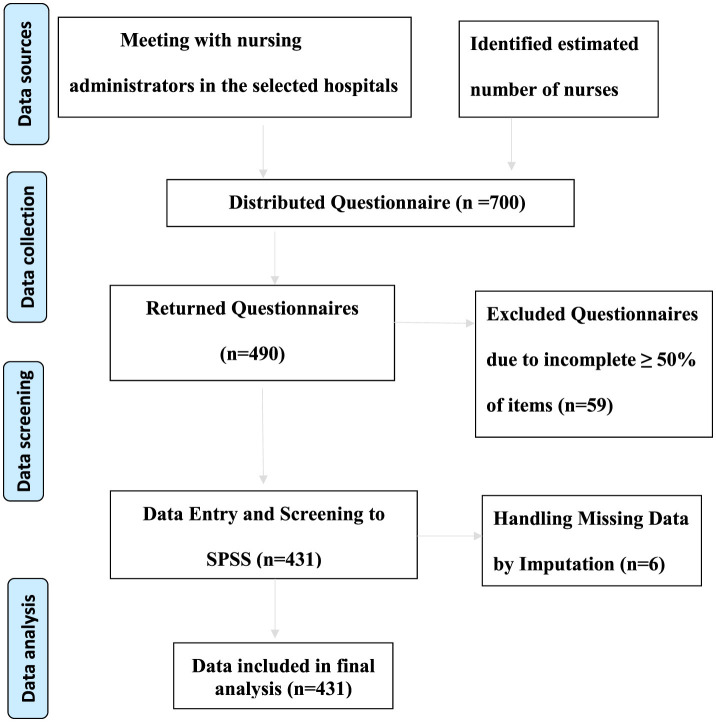
Data management flowchart.

Data were collected using a self-report instrument (from December 2022 to June 2023). The researcher interviewed the head of nursing department in the selected hospitals to know the estimated number of nurses in each hospital. While potential participants were on duty at the selected hospitals, the first researcher invited them after explaining the study, its aims, and its benefits. Interested participants were asked to sign a consent form and complete three questionnaires. The average time required to complete the questionnaires was 15 min. However, owing to the urgent nature and large workload in some departments, each potential participant was given 2 h to return the completed questionnaires. To visually represent how data were collected, processed, and analyzed in our study, a data management flow chart was included (Figure 1).

### Outcome measure

A modified version of the questionnaire developed and validated by the ILO/ICN/WHO/PSI Workplace Violence in the Health Sector Country Case Study Questionnaire in 2003 was used to measure the participants' prevalence of WPV and its' contributing factors (49). Permission to administer the survey questionnaire was obtained from the ILO Publications Bureau. The original questionnaire was written in English and included five sections focusing on personal experiences, physical and psychological aspects, and participants' opinions. This study focused on verbal and physical WPV; thus, bullying/mobbing, harassment, and racial harassment were excluded from the questionnaire. Based on the purpose of the study and after necessary adjustments were made, the questionnaire consisted of three main sections: (1) Personal and workplace data (16 items), (2) Consequences of WPV (9 items), and (3) Opinions on WPV (contributing factors and suggestions to reduce WPV that the author originated from the items based on the literature review) (13 items). A pilot study was conducted with 10% of the sample size, involving participants selected from the nursing staff. However, these participants were later excluded from the final study. The pilot aimed to evaluate the clarity, suitability, and comprehensibility of the questionnaire. The questionnaire's reliability was assessed by measuring internal consistency, which revealed a high reliability coefficient with a Cronbach's alpha of 0.82.

### Data analysis

SPSS version 28 was used to analyze the data (IBM, 2021). Data entered into SPSS after handling the missed data in six questionnaires (some items had missing data which were missing at random). Replacing the missing data occurred through imputation with a series mean (*n* = 6). Descriptive statistics were used to analyze the WPV and contributing factor results. For categorical variables, the number and percentage distributions by category were calculated.

## Results

### Demographic and work characteristics

The ages of the participants ranged from 20 to 49 years. More than half of the participants were females (*n* = 237) aged 20–29 years, married (*n* = 236), and had a bachelor's degree (*n* = 301), as shown in Table 1. Most participants (93.5%, *n* = 403) were staff members in their respective departments. Approximately 58.5% of the participants had <5 years of clinical experience (*n* = 252). Additionally, 361 participants (83.3 %) reported working with male or female patients in their respective units or departments. Furthermore, 57.3% dealt with patients in the adolescent-to-older adult age group. The highest percentage of participants (*n* = 101, 23.5%) worked in the emergency departments. Most nurses (84%) mentioned that the number of staff members in their units was between 1–5 at any given time.

**Table 1:** Demographical and work characteristics of the sample (*N* = 431).

**Characteristic**	***N* (%)**
**Gender**	
Male	194 (45%)
Female	237 (55%)
**Age group**	
20–29 years	241 (55.9%)
30–39 years	154 (35.7%)
40–49 years	36 (8.4%)
**Marital status**	
Single	187 (43.4%)
Married	236 (54.8%)
Separate/Divorce	8 (1.9%)
**Education level**	
Diploma	96 (22.3%)
Bachelor	301 (69.8%)
Master	34 (7.9%)
**Work experience**	
<5 years	252 (58.5%)
5–10 years	99 (23%)
More than 10 years	80 (18.6%)
**Number of staff in same unit**	
Alone	36 (8.4%)
1–5 staffs	362 (84%)
More than 5 staffs	33 (7.6%)
**Sex of patients in the unit**	
Male or female	70 (16.2%)
Male and female	361 (83.3%)
**Patients' age group in working area** ^a^	
Infant	210 (12%)
Newborn	210 (12%)
Child	326 (18.7%)
Adolescent	321 (18.4%)
Adult	352 (20.2%)
Older adult	324 (18.6%)
**Professions**	
Physicians	94 (21.8%)
Nurses & midwifes	247 (57.3%)
Professions allied to medicine	34 (7.9%)
Technical & administration staffs	56 (13%)
**Current position**	
Head of department	8 (1.9%)
Specialist & resident physicians	20 (4.6%)
Staffs member of department	403 (93.5%)
**Working department**	
Medical & surgical units	79 (18.3%)
Critical care units	40 (9.3%)
Emergency department	101 (23.5%)
Obstetrics& gynecology units	78 (18.1%)
Orthopedics, dialysis & operation units	38 (8.8%)
Pharmacy, dietitian, radiology& laboratory	95 (22%)
**Working in different shifts**	
Yes	392 (91%)
No	39 (9%)
**Factors impact whether or not to report a violent incident**,	
**assault, or threatening behavior at work** ^a^	
The severity of the incident	129 (19%)
Which supervisor is on shift	96 (14.1%)
Whether or not co-workers are supportive	61 (9%)
The condition of the patient	157 (23.1%)
The reporting procedure is unclear	70 (10.3%)
The purpose of reporting is unclear	39 (5.7%)
Fear of retaliation	34 (5%)
Other	94 (13.8%)
**Worrying level about violence in current workplace**	
No worries	192 (44.5%)
Low - Moderate worries	72 (16.7%)
High - Extremely worries	167 (38.7%)
**Does employer have successful program to prevent workplace**	
**violence?**	
Yes	34 (7.9%)
No	327 (75.9%)
Not sure	70 (16.2%)
**Physical violence experienced in the last 12 months**	
Yes	110 (25.5%)
No	321 (74.5%)
**Verbal violence experienced in the last 12 months**	
Yes	349 (81%)
No	82 (19%)

### Prevalence of verbal and physical WPV

A high percentage (81%) of the nurses reported experiencing verbal violence, while 25.5% reported experiencing physical violence. More than half of the nurses reported feeling worried about being attacked in the workplace (44.5%). Factors such as patient condition (23.1%) and the severity of the incident (19%) were the two most common factors affecting nurses' willingness to report a violent incident, assault, or threatening behavior. Additionally, most nurses declared the absence of a preventive program in their units or work areas (75.9%) (Table 1).

### Consequences of WPV incidents in the last 12 months

Of the 110 physically attacked nurses, 44 (40 %) reported that an investigation was conducted to determine the cause of the incident. Most of the attacked workers did not sustain any injury (70.9%), whereas the rest suffered injuries (29.1%). Twenty-nine of the injured participants took time off work after being attacked (26.4%), and the majority of sick leaves after an attack lasted for less than a week (62.1%) (Table 2).

**Table 2:** Consequences of physical WPV incidents in the last 12 months (*N* = 431).

**Consequences of physical violence**	***N* (%)**
**Action taken to investigate the causes of the incident**	
Yes	44 (40%)
No	37 (33.6%)
Don't know	29 (26.4%)
**Injuries as a result of the physical violence**	
Yes	32 (29.1%)
No	78 (70.9%)
**Time taken off from work after being attacked**	
Yes	29 (26.4%)
No	81 (73.6%)
**Duration of time taken off from work after being attacked**	
Less than one week	18 (62.1%)
1–4 weeks	7 (24.1%)
More than 4 weeks	4 (13.8%)
**This incident attacked by using weapon**	
Yes	42 (38.2%)
No	68 (61.8%)
**This incident considered as routinely conducted in workplace**	
Yes	61 (55.5%)
No	22 (20%)
Don't know	27 (24.5%)
**Consequences for the attacker** ^a^	
None	33 (14%)
Verbal warning issued	56 (23.7%)
Care discontinued	27 (11.4%)
Reported to police	47 (19.9%)
Aggressor prosecuted	73 (31%)
**Reasons for not reporting the incident** ^a^	
Not a target or witness of violence	64 (21.4%)
Not important to report	39 (13%)
Reporting never lead to change	96 (32.1%)
Not sure how to report	41 (13.7%)
No particular reason	25 (8.4%)
Didn't have a time	34 (11.4%)
**Worker**′**s satisfaction with the manner in which the**	
**physical incident was handled**	
Very dissatisfied	28 (25.5%)
Dissatisfied	22 (20%)
Moderately satisfied	17(15.5%)
Satisfied	6 (5.4%)
Very satisfied	37 (33.6%)

^a^Each participant can provide more than one answer.

Approximately 38.2% of incidents involving physical violence in the last 12 months involved the use of weapons. The most commonly reported consequences for attackers were prosecution and verbal warnings (31% and 23.7%, respectively), while discontinuing care was the least common consequence. Many nurses did not report physical incidents to others because they felt it would not lead to any change (36.2%). Lack of importance and time were the least common reasons for not reporting physical violence (13% and 11.4%, respectively). Of those who had been physically attacked, 55.5% said that these incidents were routine occurrences in the workplace. Although 33.6% of physically attacked workers were satisfied with how the incident was handled, 45.5% expressed dissatisfaction with the overall handling of the situation (Table 2).

The current study revealed that 59.6% of the nurses reported that verbal incidents were common in their workplace. The most common consequence for attackers after causing verbal violence was prosecution, with 37.7% of the incidents reported resulting in this action. Verbal warnings and reporting to the police were the next most common consequences, accounting for 24.7% of incidents. Discontinuing care is the least common consequence of these attacks (Table 2).

Approximately 50.4% of the participants stated that no action was taken to investigate the cause of verbal incidents or that they were unaware of any action being taken. More than half of those who were verbally attacked (53.8%) reported dissatisfaction with how the incident was handled (*n* = 188), whereas only 19.8% reported satisfaction. For a detailed overview of the consequences of verbal WPV (see Table 3).

**Table 3:** Consequences of verbal workplace violence in the last 12 months (*N* = 431).

**Consequences of verbal violence**	***N* (%)**
**Action taken to investigate the causes of the incident**	
Yes	173 (49.6%)
No	162 (46.4%)
Don't know	14 (4%)
**Consequences for the attacker** ^a^	
None	58 (12.5%)
Verbal warning issued	114 (24.7%)
Care discontinued	2 (0.4%)
Reported to police	114 (24.7%)
Aggressor prosecuted	174 (37.7%)
**This incident considered as routinely conducted in workplace**	
Yes	208 (59.6%)
No	73 (21.2%)
Don't know	66 (19.2%)
**Worker's satisfaction with the manner in which the**	
**incident was handled**	
Very dissatisfied	116 (33.2%)
Dissatisfied	72 (20.6%)
Moderately satisfied	92 (26.4%)
Satisfied	13 (3.7%)
Very satisfied	56 (16.1%)

^a^Each participant can provide more than one answer.

### Contributing factors beyond WPV

Table 4 shows the frequency of participants' responses regarding factors contributing to WPV. The highest level of agreement among all participants was leniency in applying penalties for violence perpetrators inside hospitals (*n* = 382, 88.6%). The second highest item was the failure to apply regulations and rules fairly in hospitals, with 84.5% of the participants agreeing (*n* = 364). Additionally, the majority of participants (83.8%) agreed that poor oversight and security in hospitals, as well as poor communication between healthcare providers and patients/families (82.6%), were contributing factors to WPV (*n* = 356). On the other hand, only 39.4% of the participants agreed that poor quality of care from staff toward patients was a contributing factor to violence in their hospitals. Furthermore, 68.2% of the participants agreed that tribal nepotism and tribal cultural control were contributing factors to WPV (Table 4).

**Table 4:** Contributing factors beyond exposure to workplace violence.

**Contributing factors**	**Agreement**	**Uncertain**	**Disagreement**
Poor communication between healthcare provider and patient/family	356 (82.6%)	37 (8.6%)	38 (8.8%)
Tribal nepotism and tribal culture control	294 (68.2%)	87 (20.2%)	50 (11.6%)
Failure to apply regulations and rules fairly in hospitals	364 (84.5%)	37 (8.6%)	30 (7%)
The leniency in the application of penalties against the perpetrators of violence inside the hospital.	382 (88.6%)	27 (6.3%)	22 (5.1%)
The feeling that violence is a means of achieving goals.	302 (83.3%)	55 (12.8%)	74 (17.2%)
Poor oversight and security in hospitals.	361 (83.8%)	29 (6.7%)	39 (9%)
Poor quality of care from staff	170 (39.4%)	109 (25.3%)	152 (35.3%)
Delay in investigations of incidents and issues related to violence in hospitals	319 (74%)	65 (15.1%)	47 (10.9%)
Favoritisms	333 (77.3%)	80 (18.6%)	18 (4.2%)
Shortage of staff	357 (82.8%)	32 (7.4%)	42 (9.7%)

### Suggestions and strategies to reduce WPV

Table 5 displays the frequency of responses to suggestions and strategies for reducing WPV. The majority of participants (95.8%) agreed that improving staff-patient communication skills would effectively reduce violence, and 89.6% stated that enhancing their competence in diagnosing and treating patients while reducing wait times would also help minimize violence. Moreover, applying strict laws and regulations to prevent family members and relatives of patients from entering areas where staff care and diagnosis are taking place could also decrease the incidence of violence. However, only 15.1% believed that assigning clear roles and responsibilities to medical workers according to their job descriptions would be effective in reducing violence. Approximately 70% of the participants suggested that if hospitals conducted annual surveys to evaluate staff and patient satisfaction and improve reporting, statistics, and violence interventions, physical and verbal attacks in the workplace could be reduced.

**Table 5:** Suggestions and strategies to reduce workplace violence.

**Suggestions and Strategies**	**Agreement**	**Uncertain**	**Dis-agreement**
Improve staff-patient communication skills	413 (95.8%)	12(2.8%)	6 (1.4%)
Improve competence in diagnosis and treatment and shorten the waiting time	386(89.6%)	31 (7.2%)	14 (3.2%)
Patient screening (to record and be aware of previous aggression behaviors)	332(77%)	71 (16.5%)	28 (6.5%)
Hospital improvements in violence reporting, statistics, and interventions	300(69.6%)	81 (18.8%)	50 (11.6%)
Police officers stationed in the hospital	340(78.9%)	54 (12.5%)	37 (8.6%)
Develop annual surveys to evaluate staff and patient satisfactions	301(69.8%)	80 (18.6%)	50 (11.6%)
Strict laws and regulations to prevents families and relatives of patient to attain in the scene of staff–patient care and diagnostic areas	380 (88.2%)	26 (6%)	25 (5.8%)
Install cameras onwards, keep work areas bright by using lights at night	319 (74%)	52 (12.1%)	60 (13.9%)
Restricted entry of the public	361(83.9%)	41(9.5%)	29 (6.7%)
Enact workplace violence legislation	378 (87.7%)	51(11.8%)	2 (0.5%)
Develop violence prevention guidelines and plans	325 (75.4%)	67(15.5%)	39 (9%)
Correct perspective and reports by media, promote respect of medical workers	339 (78.7%)	71 (16.5%)	21 (4.9%)
Clear roles and responsibilities for medical workers committed to job descriptions	322 (74.7%)	44 (10.2%)	65 (15.1%)

## Discussion

One of the main findings was that 44% of nurses who experienced physical violence reported that an investigation was conducted to determine the cause of violence. However, no action was taken against the perpetrators; instead, staff members were informed of the problem. This aligns with the contributing factors identified in the present study. Previous studies have shown that one-third of nurses believe that reporting incidents of violence will not lead to any change in the current situation (8, 14, 15). Inadequate assertive policies are believed to be responsible for this situation, as supported by the results of our study. The absence of clear policies and protocols to address this issue is one of the factors contributing to WPV against nurses in Jordan. Without proper guidelines and procedures, healthcare institutions face difficulties in preventing and managing violent incidents effectively. Comprehensive policies can provide a framework for prevention, reporting, and appropriate disciplinary actions (3, 43, 44).

Another significant finding of the current study was that more than half of the participants considered physical violence to be a routine incident in the workplace. This suggests that there are few policies or actions aimed at reducing WPV (18, 28). Poor management or admission procedures, a lack of rules and penalties, and other factors can contribute to violence becoming a routine occurrence (43). In addition, the current study showed that male workers were more exposed to violence than female workers. This can be attributed to certain cultures that teach males to believe that they are socially superior to women and that impulsive actions are necessary to be considered a “true man.” (50). These ideas of masculinity may contribute to male nurses' increased exposure to violence. Gender dynamics also play a role in WPV against nurses in Jordan. Female nurses may face a higher risk of violence because of gender-based discrimination and stereotypes (51). A multifaceted approach is required to address this issue. Empowering female nurses, promoting gender equality in the workplace, and fostering a supportive environment that values diversity and inclusivity are some measures to tackle WPV against nurses (21, 44).

Studies have supported the idea that WPV can have physical, verbal, and other negative consequences. This can also result in many factors contributing to violence and various suggestions for limiting it. WPV can cause delays in effective communication, impaired peer relations, poor concentration at work, and prevent nurses from providing safe and effective care to patients (17, 24, 32). It can also lead to failure to raise safety concerns and seek assistance, resulting in delayed care (52). Another significant finding was that most participants did not take time off work after being attacked. However, exposure to violence can lead to job dissatisfaction, increased staff turnover/attrition rate, and errors in treatments or medications (15, 33, 37, 53). Workplaces can be challenging environments for workers, particularly when they are overloaded and lack knowledge about handling and reporting violence. Inadequate staffing levels and heavy workloads put a lot of pressure on nurses, which can create an environment that is conducive to WPV (33, 37, 53). When nurses are overburdened, stressed, and unable to meet patient needs adequately, tensions can rise and frustration can escalate, leading to violent outbursts. To mitigate WPV, it is important to address staffing issues and ensure that workloads are manageable.

Approximately 31% of the respondents reported that verbal warnings were issued as a consequence of the attacker, which may have led to recurrent violent behavior. Another 23.7% of participants reported that the aggressor was prosecuted. Poor management and leadership practices can contribute to WPV among nurses in Jordanian hospitals. This can manifest in various forms such as lack of support and communication, failure to address and respond to incidents of violence, and a hierarchical culture that does not prioritize nurses' wellbeing (13, 25, 54). Hospital management must foster a supportive and inclusive work culture, provide adequate resources and training for managers, and ensure that nurses have channels to report incidents of violence without fear of retribution (55). In some Jordanian hospitals, a culture of violence and acceptance of aggression exists, which contributes to WPV against nurses (27, 42, 56). This culture can stem from various factors, such as a lack of consequences for aggressive behavior, normalization of verbal or physical abuse, and a hierarchical structure that perpetuates power imbalances (27). Addressing this issue requires collective effort, including strict enforcement of policies against WPV and the promotion of a culture of respect and professionalism (24).

It has been reported that verbal violence is as common as physical violence in Arab countries (57). However, many people who experience verbal violence do not report it because of fear of negative consequences or inadequate reporting procedures. In addition, health care providers, especially female ones, may not know how to handle or defuse violent situations (12). This can be influenced by cultural norms that undermine the authority and professionalism of nurses, leading to disrespectful behavior and aggression (27). To address this issue, awareness must be raised and nursing as a critical profession in healthcare must be promoted to challenge negative perceptions and foster a culture of appreciation and respect.

The findings reveal that the main contributing factor to WPV in hospitals, beyond exposure, is leniency in the application of penalties against perpetrators of violence inside the hospital. This is consistent with the results of previous studies (15, 27, 39, 42, 44). Therefore, it is essential to implement assertive policies and rules in hospitals to protect health care providers and enable them to provide care while feeling safe. Another important contributing factor is the failure to fairly apply regulations and rules in hospitals, which affects care delivery and contradicts human rights. Poor oversight and security in hospitals can worsen violence, which was reported by 83.8% of participants and is consistent with other studies (11, 27). Inadequate security measures and infrastructure in Jordanian hospitals can make nurses vulnerable to WPV. These include limited security personnel, insufficient surveillance systems, and poorly designed facilities that do not prioritize the safety of healthcare workers. Improving security measures, increasing the presence of security personnel, and investing in proper infrastructure are essential steps toward creating a safer work environment for nurses.

We summarize our main findings on effectively reducing violence against nurses in peripheral healthcare settings by examining the major strategies and recommendations suggested by the participants. 95.8 of the respondents, 95.8% stated that improving staff-patient communication skills is crucial. This can be interpreted as nurses being unable to understand and respond effectively to patients' needs. Miscommunication between care providers (nurses) and care seekers (patients and their families) can often lead to angry reactions from patients and their escorts (15, 27, 42, 58). Improving communication skills reduces verbal and physical violence (46). A total of 89.6% of participants recommended improving their competencies in diagnosis and treatment to reduce waiting times. Previous research has found that patients' and family members' anger can stem from a lack of competence during treatment (55). Of the respondents, 88.2% suggested introducing strict laws and regulations to prevent relatives from interfering in staff-patient care and restricting public entry to minimize the impact of violence. Most nurses (83.9%) suggested that effective communication and teamwork are crucial for creating a safe and supportive work environment. However, communication breakdowns and lack of teamwork can contribute to WPV. Poor communication among staff members, between healthcare professionals and patients, or inadequate conflict resolution skills can escalate tensions and increase the likelihood of violence (33, 59). Encouraging open dialogue, fostering respectful communication, and promoting teamwork are essential for minimizing WPV in Jordanian hospitals.

Nurses require effective coping strategies and support systems to deal with WPV. These include training programs equipping nurses with de-escalation techniques, self-defense training, and mental health support services (33). The Occupational Health and Safety Act (2019) highlights the pivotal role of occupational health services in mitigating verbal and physical violence against nurses. In countries like Jordan, where peripheral hospitals often face resource shortages, understaffing, and heavy patient loads, the risk of workplace violence is elevated (28, 56). To address these challenges, occupational health services offer targeted training for nurses in areas such as de-escalation, stress management, and conflict resolution, which are critical in resource-constrained environments (60). Furthermore, occupational health programs can advocate for improved staffing, structured breaks, and mental health support through counseling services (61, 62). Thus, creating a supportive work environment that encourages open communication, provides access to counseling services, and promotes peer support can also help nurses navigate the emotional and psychological impact of WPV (63, 64).

In Jordan, several studies have also underscored the importance of integrating preventive strategies and education to enhance occupational health and safety for healthcare workers. Ashour and Hassan recommend incorporating safety training and collaboration as integral components of safety management across different organizational environments (65). Similarly, Al-Natour et al., discuss strategies employed by nurses to manage workplace violence but note that these strategies lack specific education or training components (27). Rababah further emphasizes the need for adopting occupational health and safety standards in various sectors, aiming for comprehensive quality benchmarks that ensure the highest safety levels (66). Through regular risk assessments and preventive health screenings, occupational health services foster safer working conditions, even in under-resourced settings, and promote a culture of safety and wellbeing, ultimately reducing workplace violence and ensuring nurses feel protected in their roles (67, 68).

### Limitations

However, this study has some limitations, such as the use of a self-reported questionnaire that could be subject to self-reporting bias, recall bias, or underreporting of incidents. Additionally, this study focused on peripheral (rural) hospitals, which may not fully represent the situation in other hospitals. Additionally, the study examined verbal and physical WPV, while items for bullying, mobbing, harassment, and racial harassment were excluded, which could be related to an inadequate capture of the full scope of the problem. Acknowledging these limitations is vital for maintaining the credibility of the study and providing a clear understanding of its scope.

### Implications

To address WPV in Jordanian hospitals, it is crucial to implement appropriate policies and preventive measures. This includes developing and enforcing clear policies against WPV, providing regular training sessions on violence prevention for all healthcare staff, and establishing reporting mechanisms that ensure the anonymity and safety of reporting incidents. In addition, hospitals should collaborate with law enforcement agencies to ensure swift responses to violence.

Education and training are essential to address WPV. Creating awareness among healthcare professionals, patients, and the general public about the impact of WPV and the importance of respect and professionalism is crucial. Training programs should be designed to teach nurses effective communication and de-escalation techniques, and how to report and document incidents of violence. By investing in education and training, hospitals can create safer and more supportive environments for nurses.

## Conclusions

WPV against nurses in Jordanian hospitals is a serious issue that affects not only the wellbeing of nurses, but also the quality of patient care. This study highlights the various consequences and contributing factors to WPV, emphasizing the need for immediate action and intervention. To create a safe, respectful, and supportive environment for nurses, comprehensive policies must be implemented, organizational practices improved, and sociocultural norms addressed. It is important for all stakeholders, including healthcare institutions, policymakers, and society, to prioritize the safety and wellbeing of nurses. This ensures that they continue to provide high-quality care without fear of violence or aggression. Mitigating WPV against nurses requires a comprehensive, multifaceted approach. This approach should include the implementation of policies and protocols aimed at preventing incidents, improving security measures and infrastructure, providing training and education on violence prevention and de-escalation techniques, promoting a supportive organizational culture, and addressing sociocultural factors that contribute to violence. By collectively addressing these factors, we can create safer and more respectful working environments for nurses in Jordanian hospitals.

## Data Availability

The original contributions presented in the study are included in the article/supplementary material, further inquiries can be directed to the corresponding author.
